# Imipramine Inhibits Chikungunya Virus Replication in Human Skin Fibroblasts through Interference with Intracellular Cholesterol Trafficking

**DOI:** 10.1038/s41598-017-03316-5

**Published:** 2017-06-09

**Authors:** Sineewanlaya Wichit, Rodolphe Hamel, Eric Bernard, Loïc Talignani, Fodé Diop, Pauline Ferraris, Florian Liegeois, Peeraya Ekchariyawat, Natthanej Luplertlop, Pornapat Surasombatpattana, Frédéric Thomas, Andres Merits, Valérie Choumet, Pierre Roques, Hans Yssel, Laurence Briant, Dorothée Missé

**Affiliations:** 1 Laboratoire MIVEGEC, UMR 224 IRD/CNRS/UM1, Montpellier cedex 5, 34394 France; 2 Centre d’Étude d’Agents Pathogènes et Biotechnologies pour la Santé, CNRS-UMR 5236/UM, Montpellier cedex 5, 34293 France; 3 Department of Microbiology and Immunology, Faculty of Tropical Medicine, Mahidol University, Bangkok, 10400 Thailand; 4 Department of Pathology, Faculty of Medicine, Prince of Songkla University, Songkla, 90110 Thailand; 5 Institute of Technology, University of Tartu, Tartu, 50411 Estonia; 6 Environment and Infectious Risks Unit, Institut Pasteur, Paris, 75015 France; 7 CEA, iMETI, Division of Immuno-Virology, Université Paris Sud, Orsay, France; 8 Center for immunology of viral infections and autoimmune diseases Inserm, UMR 1184, Fontenay-aux-Roses, 91190 France; 9 Centre d’Immunologie et des Maladies Infectieuses, Inserm, U1135, Sorbonne Universités, UPMC, APHP Hôpital Pitié-Salpêtrière, Paris, 75013 France

## Abstract

Chikungunya virus (CHIKV) is an emerging arbovirus of the *Togaviridae* family that poses a present worldwide threat to human in the absence of any licensed vaccine or antiviral treatment to control viral infection. Here, we show that compounds interfering with intracellular cholesterol transport have the capacity to inhibit CHIKV replication in human skin fibroblasts, a major viral entry site in the human host. Pretreatment of these cells with the class II cationic amphiphilic compound U18666A, or treatment with the FDA-approved antidepressant drug imipramine resulted in a near total inhibition of viral replication and production at the highest concentration used without any cytotoxic effects. Imipramine was found to affect both the fusion and replication steps of the viral life cycle. The key contribution of cholesterol availability to the CHIKV life cycle was validated further by the use of fibroblasts from Niemann-Pick type C (NPC) patients in which the virus was unable to replicate. Interestingly, imipramine also strongly inhibited the replication of several *Flaviviridae* family members, including Zika, West Nile and Dengue virus. Together, these data show that this compound is a potential drug candidate for anti-arboviral treatment.

## Introduction

Chikungunya virus (CHIKV) is an arbovirus transmitted by the genus *Aedes spp*. that causes chikungunya fever, an infectious disease characterized by myalgia, joint pain, rash, and intense asthenia disease^1, 2^. A common long-term complication in many patients is severe, long-lasting, debilitating arthralgia that may persist for months or even years after infection, giving sense to the name Chikungunya, meaning “the disease that bends up the joints”, coined by the Makonde people in Tanzania, where the virus was first recognized in 1952^3, 4^. Whereas initially only sporadic outbreaks of chikungunya fever have been reported on the African continent and in Southeast Asia, the spread and propagation of the vector, in particular *Aedes albopictus*, over the past decade has resulted in millions of infections in more than 50 countries, not only in tropical, but also temperate regions^5, 6^. Currently, there is no licensed vaccine or antiviral treatment available to prevent or treat CHIKV infection and this arbovirus thus clearly presents a global threat for public health.

CHIKV has icosahedral-shaped enveloped virions (approximately 70 nm in diameter) and belongs to the alphavirus genus within the *Togaviridae* family^7, 8^. Its genome is composed of a single positive stranded RNA molecule of 12 kb, organized in two open reading frames (ORFs). The 5′-terminal ORF encodes four nonstructural proteins (nsP1-4) that form a replication complex involved in the synthesis of genomic RNA, as well as a subgenomic RNA^9^. The latter is translated, via the second ORF, into six structural proteins (capsid (C) protein, E1, E2, and E3 glycoproteins and 6 K/TF proteins) that are all involved in the assembly of viral particles, their attachment and entry into target cells^10–12^.

CHIKV is internalized into human target cells as a result of receptor-mediated endocytosis that involves both clathrin-dependent and independent mechanisms^13, 14^ and is subsequently delivered to early endosomes from which the virus capsid is released into the cytosol through the formation of fusion pores. The latter process is triggered by the low-pH environment in the endosome that induces an irreversible change in glycoprotein conformation and a dissociation of the E2/E1 heterodimers, followed by E1 trimerizations^11^. The viral RNA synthesis occurs in replication complexes located in proximity of plasma membrane^15^. The E1 and E2 proteins are translated at the endoplasmic reticulum, transported through the Golgi, processed in the trans Golgi network, and finally transported to the plasma membrane where virus budding occurs^16^.

Since lipid membranes do not mix spontaneously, the fusion and budding process is not only energy-dependent, but also conditioned by the lipid composition of both the viral envelope and host cell membrane, in particular by the presence of cholesterol and sphingolipids. Therefore, abnormalities in cholesterol metabolism may affect the rate and efficiency of the production of CHIKV virions at multiple steps. Indeed, depletion of cholesterol in competent cells using methyl β-cyclodextrin was reported to strongly inhibit CHIKV infection^14^, which is in line with the generally accepted idea that cholesterol is required for optimal cell membrane fusion, viral replication and budding of prototype alphaviruses, such as Sindbis virus^17^ and CHIKV^18^, as well as other arboviruses, including West Nile virus (WNV), Japanese encephalitis virus and dengue virus (DENV)^19–22^.

Cholesterol is an important biological molecule in membrane structures and is acquired by mammalian cells via *de novo* synthesis, as well as from dietary sources by receptor-mediated endocytosis. Both synthesized and dietary cholesterol are transported through the circulation in low density lipoproteins (LDLs). This is also the case for cholesteryl esters, the form in which cholesterol is stored in cells. The LDLs are then delivered to the late endosomal and lysosomal (LE/Ls) compartments, followed by the hydrolyzation of cholesteryl esters by acid lipase and the subsequent release of free cholesterol into the cytoplasm. Unesterified cholesterol exits the LE/Ls through a mechanism that is dependent on the activity of the Niemann-Pick C (NPC) type 1 and type 2 proteins, well-known for their role in the intracellular trafficking of cholesterol^23, 24^. Their importance is underscored by the observation that mutations in either one of the NPC1 or NPC2 genes may lead to loss of function of these two endosomal membrane proteins, resulting in the development of NPC disease, a rare, but fatal, autosomal recessive, lysosomal storage disorder, characterized by an accumulation of cholesterol, sphingomyelin, sphingosine, as well as the GM1 and GM3 gangliosides within the LE/Ls^25^.

The molecular phenotype of NPC disease can be mimicked by the treatment of cells with the class II cationic amphiphilic compounds U18666A (3-β-[2-(diethylamine)ethoxy] androst-5-en-17-one) or imipramine^26, 27^ that block the exit of cholesterol from the LE compartment and its subsequent transfer to the cell membrane. U18666A also inhibits cholesterol biosynthesis by inhibiting oxidosqualene cyclase and desmosterol reductase^28, 29^.

In the present study, we have investigated the capacity of two class II cationic amphiphilic drugs to inhibit CHIKV replication, through the interruption of cholesterol trafficking, in primary human epidermal fibroblasts, an important constituent of the skin, which is considered to be the primary entry site of the virus. The results demonstrate the therapeutic potential of such drugs.

## Results

### Inhibition of cholesterol trafficking inhibits CHIKV replication in human skin fibroblasts

**Figure 1:**
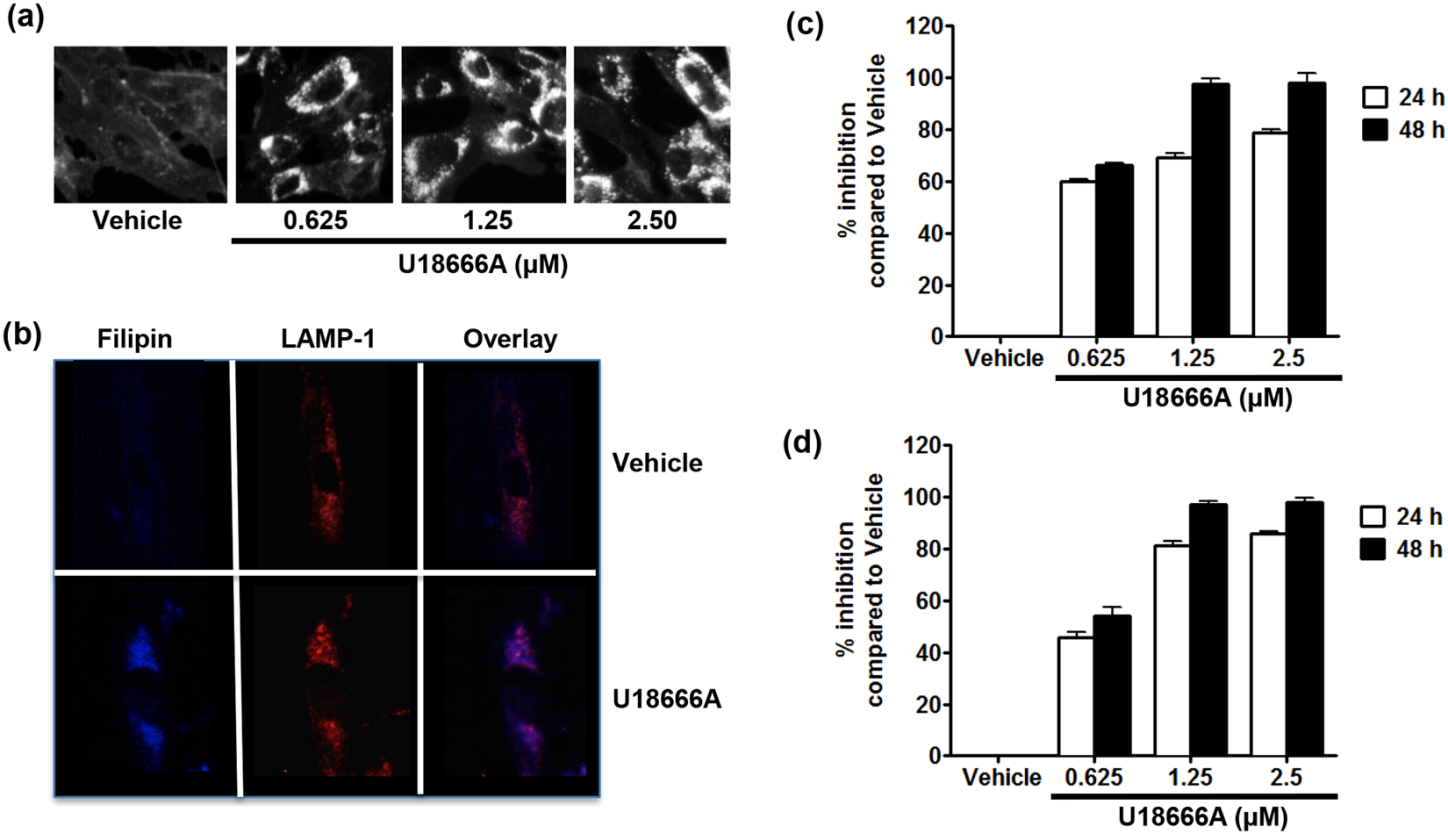
U18666A causes accumulation of cholesterol in the late endosome/lysosome and inhibits CHIKV replication in human skin fibroblasts. (**a**) Human skin fibroblasts were treated either by water (vehicle) or 0.625, 1.25 and 2.5 μM of U18666A for 24 h before fixation and labeling by filipin. (**b**) Human skin fibroblasts were treated with vehicle or 2.5 μM of U18666A for 24 h before fixation and labeling by filipin (blue) or LAMP-1 (Red). Colocalization of cholesterol and LAMP-1 are shown in violet. (**c** and **d**) Human skin fibroblasts were treated with vehicle or U18666A for 24 h before infection with CHIKV La Réunion strain (MOI 1). After 24 and 48 h, virus RNA and infectious virus production were measured by real time RT-PCR and plaque assay, respectively. Inhibition at the presence of vehicle was set as “0” and percentage of inhibition at the presence of inhibitor was calculated using formula [1 − (I/V)]*100 where V and I designate experimental values (RNA copy numbers or plaque numbers) at the presence of vehicle and inhibitor, respectively. The data represent mean ± SD from three independent experiments.

Cholesterol has been shown to play an important role in viral infection of permissive cells. In order to examine whether CHIKV replication was dependent on the accumulation of intracellular cholesterol, the human skin fibroblast cell line HFF1 was pre-treated with vehicle or U18666A for 24 h before infection with CHIKV La Réunion strain. Treatment of HFF1 cells with increasing concentrations of U18666A resulted in a dose-dependent accumulation of intracellular cholesterol in the LE/Ls compartment, as demonstrated by 100% of colocalization of the fluorescent, high affinity cholesterol-binding, chemical filipin and the LE/Ls marker LAMP-1 (Fig. 1a,b). U18666A did not have any cytotoxic effects, even at the highest concentrations used (Supplementary Fig. S1a). Pretreatment of the cells for 24 h with U18666A to allow the accumulation of cholesterol, prior to infection by CHIKV La Réunion strain for 24 and 48 h, resulted in a dose-dependent inhibition of viral replication. The effect of drug is more pronounced at the 48 h time point, as shown by a decrease in viral RNA copy numbers in the infected cells, reaching close to 97.75% at the highest concentration of the compound (Fig. 1c and Supplementary Fig. S2a). Coherently, a dose-dependent decrease in the production of viral particles was also observed (Fig. 1d Supplementary Fig. S2b).

**Figure 2:**
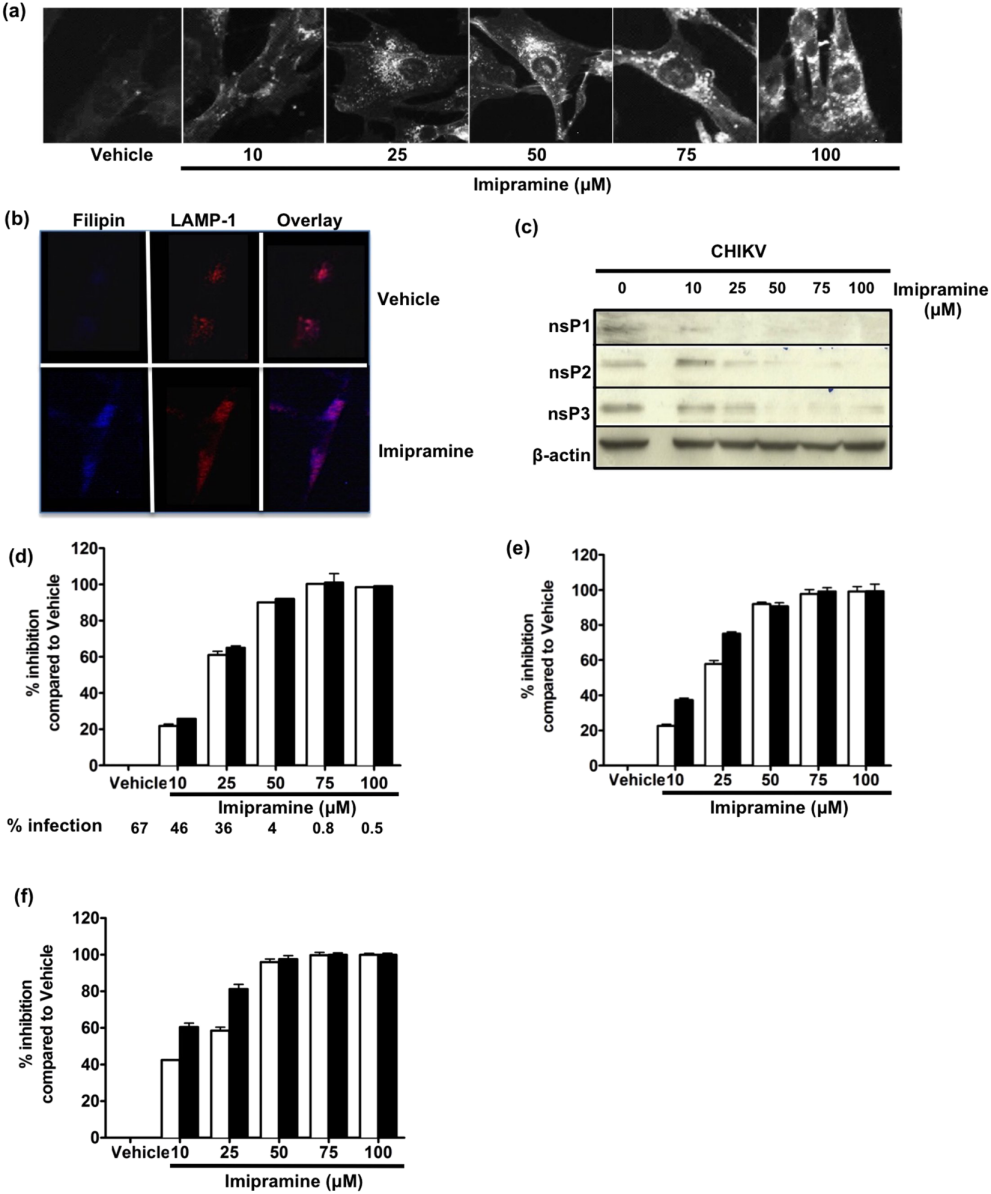
Imipramine causes accumulation of cholesterol in the late endosome/lysosome and inhibits CHIKV replication in human skin fibroblasts. (**a**) Human skin fibroblasts were treated either with PBS (vehicle) or 10, 25, 50, 75 and 100 μM concentrations of imipramine for 24 h before fixation and labeling by filipin (for a clear outline the color was changed to black and white). (**b**) Human skin fibroblasts were treated by vehicle or 75 μM of imipramine for 24 h before fixation and labeling by filipin (blue) or LAMP-1 (Red). Colocalization of cholesterol and LAMP-1 were shown in violet. (**c**–**i**) Human skin fibroblasts were pretreated by vehicle or indicated concentrations of imipramine for 2 h before exposed to CHIKV (MOI 1) La Réunion (24 h (white bar) and 48 h (black bar)) strain (imipramine maintained throughout the infection). (**c**) Infected cells were lysed with RIPA and analysed by immunoblotting against nsP1, nsP2, nsP3 and β-actin. The samples were derived from the same experiment and blots were processed in parallel. Reduction of percentage of CHIKV infected cells (**d**), inhibition of virus RNA synthesis (**e**) and infectious virus productions (**f**) were quantified by flow cytometry, real time RT-PCR and plaque assay, respectively. Percentage of inhibition was calculated as for Fig. 1. The data represent mean ± SD from three independent experiments.

Imipramine, a cationic hydrophobic amine, induces, like U18666A, lysosomal accumulation of numerous lipid species, including cholesterol^30^. Because of its general clinical use in humans for more than 60 years^31^, this FDA-approved, antidepressant drug was chosen to further investigate the consequences of perturbation of intracellular cholesterol trafficking on the replication of CHIKV. Imipramine was first evaluated for its capacity to induce a lysosomal lipid storage disease phenotype. Similar to U18666A, treatment of HFF1 cells with increasing doses of imipramine had no effect on the viability of the cells (Supplementary Fig. S1b) and also induced a dose-dependent intracellular accumulation of cholesterol starting at 25 µM (Fig. 2a). Imipramine treatment, at a concentration of 75 µM, was also found to result in 100% of colocalization of filipin and LAMP-1 in the LE/Ls compartment (Fig. 2b). Next, the effect of imipramine on the replication of CHIKV belonging to the East/Central/South African genotype (La Réunion isolate) was analyzed. Treatment of HFF1 cells with imipramine at concentrations ranging from 0 to 100 µM, from 2 h prior to infection to the end point of the experiment resulted in a dose-dependent decrease of numbers of CHIKV infected cells (Fig. 2d). Detectable inhibition was observed already at 10 µM whereas a complete inhibition of replication was observed at a concentration of 100 µM of imipramine (Fig. 2e and Supplementary Fig. S3a). The dose-dependent reduction in the release of infectious CHIKV particles was furthermore confirmed by plaque assay (Fig. 2f and Supplementary Fig. S3b). Finally, using Western blotting analysis the presence of nsP1, as well as nsP2 and nsP3, was no longer detectable at concentrations of ≥25 and ≥50 µM imipramine, respectively (Fig. 2c). Taken together, these data demonstrate that both U18666A and imipramine, two drugs that redirect and sequestrate cholesterol to the LE/Ls compartment, inhibit the replication of various CHIKV isolates in human skin fibroblasts.

### Imipramine inhibits both fusion and RNA replication steps following CHIKV infection of human skin fibroblasts

**Figure 3:**
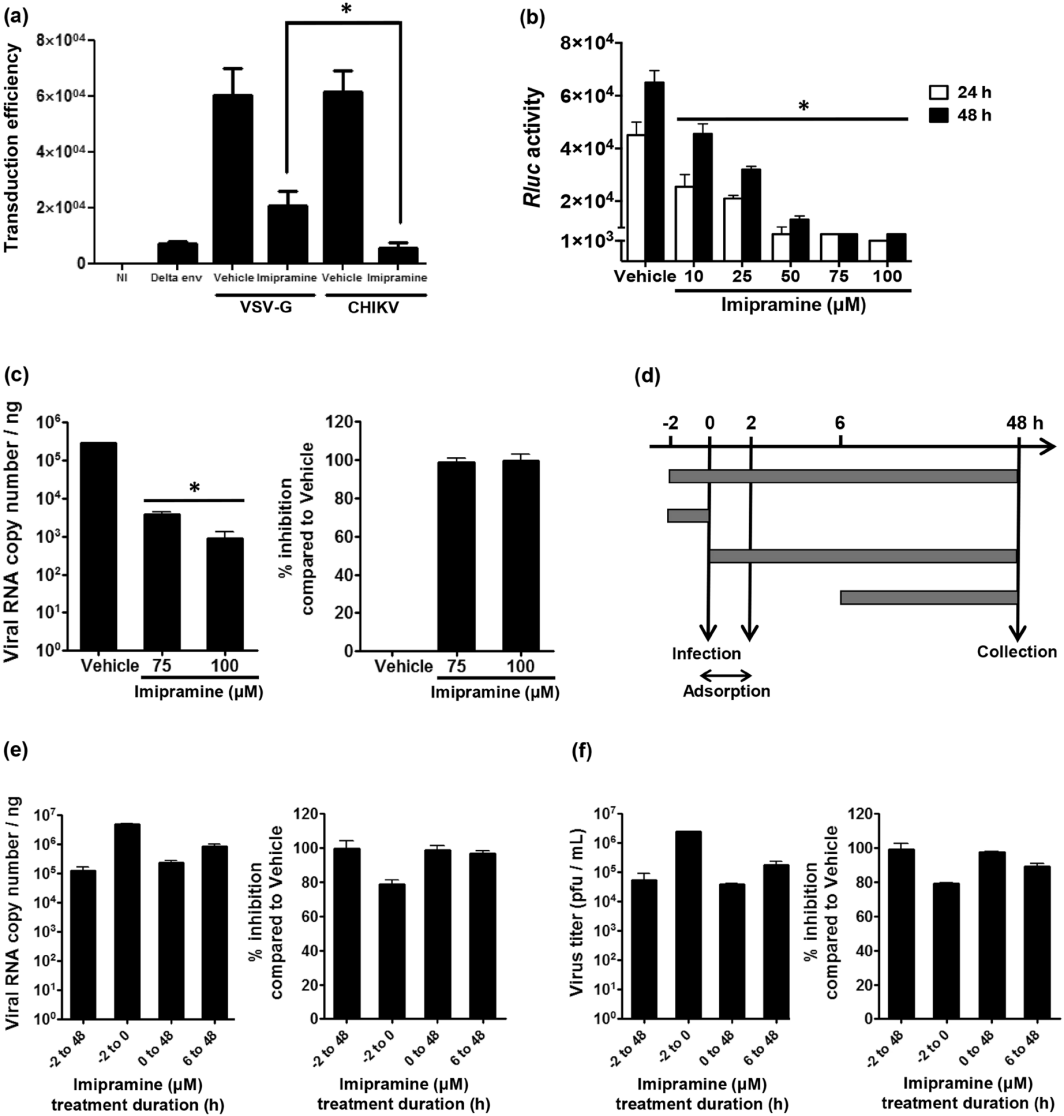
Imipramine exerts its inhibitory effects at different stages of the viral life cycle. (**a**) Human primary skin fibroblasts were either treated with 75 µM imipramine or with PBS (vehicle) for 24 h and then challenged with CHIKV Env- or VSV-G-pseudotyped HIV particles. HIV particles devoid of envelope were used to monitor potential pseudo-transduction events. Transduction of the cells was monitored by quantification of GFP expression in the cells after 48 h. Values are normalized according to protein contents of the cell extract and correspond to the mean of triplicate ± SD. NI is for non-transduced cells. *p < 0.05. (**b**) Stable transfected Huh-7 cells with CHIKV-NCT replicon were treated with different concentrations of imipramine. After 24 and 48 h, treated cells were measured for the Renilla Luciferase (*Rluc*) activity. Vehicle–treated (PBS) cells were used as control (“0”) concentration. Relative *Rluc* activity expressed by the CHIKV replicon represents CHIKV’s RNA replication. Values are normalized according to protein contents of the cell extract and correspond to the mean of triplicate ± SD. (**c**) Results of RT-PCR analysis of viral RNA copy numbers in vehicle and imipramine treated cells. Percentage of inhibition (right panel) is calculated as described for Fig. 1. The data represent mean ± SD from three independent experiments. *p < 0.05 when compared to cells treated with vehicle. (**d**) Schematic presentation of the time-of-addition assay. Human skin fibroblasts, treated as shown with 10 µM, 25 µM, 50 µM, 75 µM or 100 µM of imipramine, were infected with CHIKV La Réunion strain for 48 h. (**e**) Inhibition of virus RNA synthesis by 75 µM of imipramine was quantified using real time RT-PCR. Viral RNA copy numbers, obtained under different conditions, are shown on the left panel; right panel shows achieved percentage of inhibition calculated as for Fig. 1. (**f**) Inhibition of infectious virus production by 75 µM of imipramine was analyzed using plaque assay. Virus titers, obtained under different conditions, are shown on the left panel; right panel shows achieved percentage of inhibition calculated as for Fig. 1. Data for all used concentrations is shown on Supplemental Figs S4 and S5.

In order to determine at which step during the viral cycle imipramine exerts its antiviral activity, its effects on the fusion and RNA replication steps of CHIKV infection were evaluated. HFF1 cells pre-treated for 24 h with imipramine were transduced with CHIKV envelope-pseudotyped HIV-like particles (VLP)^32^ permitting to specifically evaluate CHIKV fusion events. At a concentration of 75 µM, imipramine inhibited the capacity of the CHIKV-VLPs to transduce HFF1 cells by ~85%. Vesicular Stomatitis Virus (VSV) G glycoprotein pseudotyped particles, transduced in these cells and used as a positive control, were also sensitive to imipramine inhibition albeit this effect was significantly lower than that observed for CHIKV-pseudotypes with a reduction of about 65% (Fig. 3a). Because cholesterol perturbators have no effect on early HIV replication events, imipramine therefore likely inhibits the CHIKV fusion/entry step^33^.

The effect of imipramine on the post-entry step of CHIKV infection was tested in the Huh7-CHIKV replicon cell line that enables to specifically measure viral RNA replication. Imipramine did not have any cytotoxic effects, even at the highest concentrations used (Supplementary Fig. S1c). The results show a dose-dependent decrease in *RLuc* activity which is directly proportional to CHIKV replication, with a maximal inhibition observed at doses of imipramine exceeding 75 µM (Fig. 3b). RT-PCR analysis confirmed that this was because of (due to a) reduction in viral RNA copy numbers (Fig. 3c). Moreover, cholesterol was found to colocalize with LAMP-1 in this cell line in the presence of imipramine (Supplementary Fig. S1d).

The effect of imipramine on CHIKV replication was further analyzed using a time-of-addition assay (Fig. 3d); this experiment was carried out in HHF cells using high (10) MOI infection with the La Réunion isolate of CHIKV. Under all conditions imipramine caused a concentration-dependent inhibition of CHIKV RNA replication and virion production (Supplemental Figs S4 and S5). Pre-treatment of cells with imipramine for 2 h caused clear, albeit relatively mild, reduction of CHIKV replication (Fig. 3e,f). Accordingly, inhibition achieved following a 2 h pre-treatment was somewhat more prominent than that observed in the absence of pre-treatment (Fig. 3e,f), thus indicating that imipramine affected the early stages of CHIKV infection. Importantly however, inhibition was reduced, but not completely lost, if imipramine was added as late as 6 hpi (Fig. 3e,f). Together, these data demonstrate that imipramine exerts its inhibitory effects at at least two different stages of the CHIKV infection cycle.

### NPC proteins are crucial for CHIKV replication

**Figure 4:**
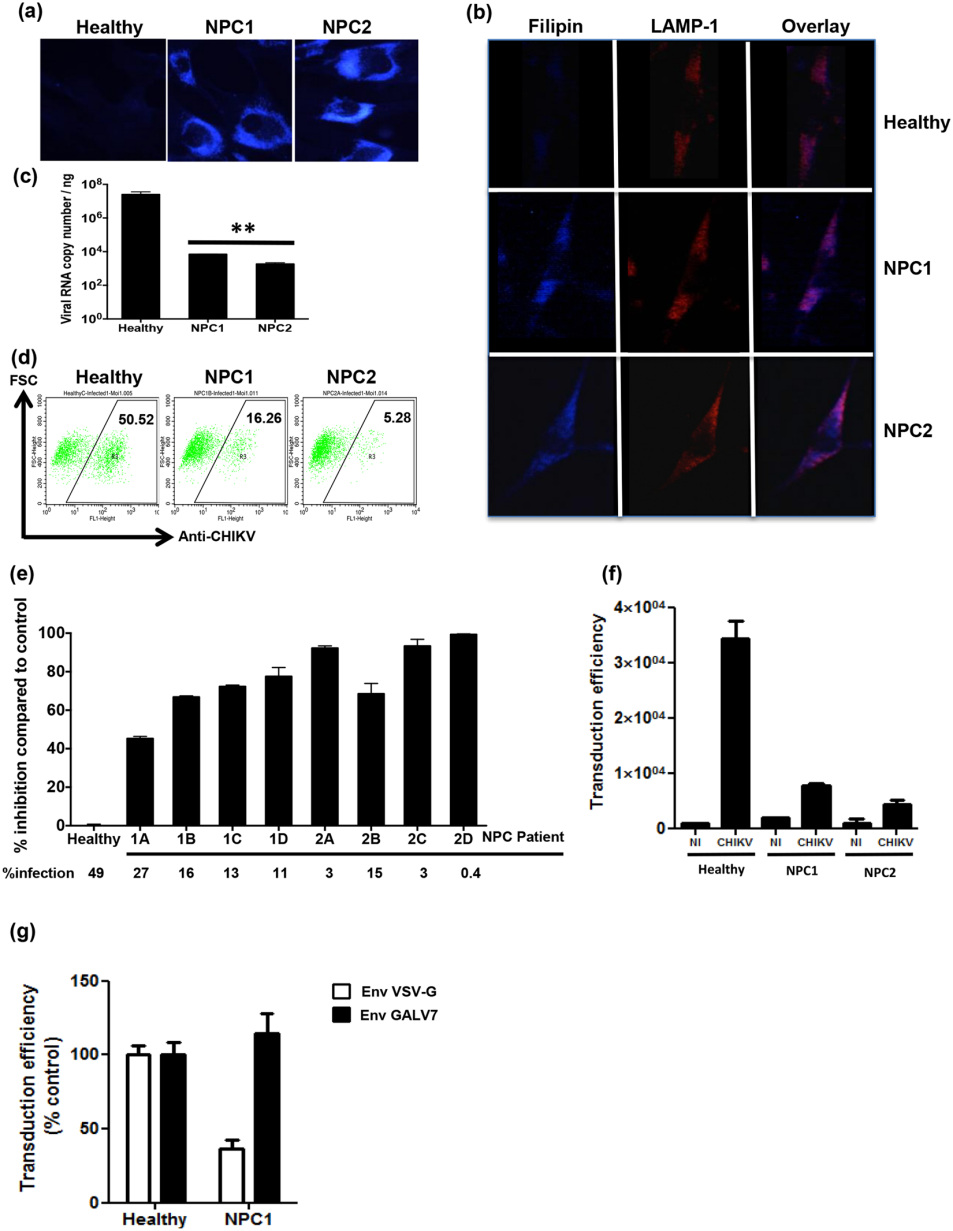
NPC1 and NPC2 proteins are crucial for CHIKV replication. (**a**) The phenotypes of NPC-deficient cells were confirmed by filipin and cholesterol accumulation examined by fluorescence microscope. (**b**) Healthy, NPC1- and NPC2-deficient cells were fixed and labeled with filipin (blue) or LAMP-1 (red). Colocalization of cholesterol and LAMP-1 are shown in violet. (**c** and **d**) Healthy, NPC1- and NPC2-deficient cells were infected with CHIKV La Réunion strain (MOI 1). After 24 h, the production of viral RNA and production of virus structural proteins were measured by real-time RT-PCR and FACS analysis, respectively. The data represent mean ± SD from three independent experiments. **p < 0.01 when compared to healthy. (**e**) Healthy, NPC1- and NPC2-deficient cells were infected with CHIKV La Réunion strain (MOI 1). After 24 h, percentage of CHIKV infected cells was measured by flow cytometry; reduction of percentage of CHIKV infected cells compared to cells from healthy control is shown. (**f**) Primary fibroblasts derived either from healthy individuals or from patients with NPC1 or NPC2 deficiencies were challenged with HIV particles pseudotyped either with CHIKVenvelope glycoproteins. Transduction of the cells was monitored by quantification of GFP in the cells after 48 h. Values are normalized according to protein content in the samples and are the mean of triplicated ± SD. NI is for non transduced cells. (**g**) Normal skin fibroblasts or NPC1 deficient cells were transduced by VSV-G- or GALV-pseudotyped particles and processed as in (**f**).

Nieman-Pick type C disease, a hereditary neurovisceral disorder, impairs egress of cholesterol from the LE/Ls compartment resulting in intracellular cholesterol accumulation^34^. As U18666A and imipramine mimic a NPC-deficient phenotype, we followed an alternative approach to highlight the consequence of intracellular cholesterol accumulation on CHIKV replication, using human fibroblasts deficient in NPC1 or NPC2 proteins. As expected, NPC-deficient fibroblasts showed an intracellular accumulation of cholesterol, in contrast to fibroblasts obtained from a healthy donor, as revealed by staining with filipin (Fig. 4a). Moreover, filipin staining colocalized with that of LAMP-1 (100% amount of colocalization), demonstrating that cholesterol accumulated specifically in the LE/Ls compartment of these cells (Fig. 4b). Absence of NPC proteins resulted in a strong decrease of CHIKV La Réunion-infection, as shown by RT-qPCR and flow cytometry (Fig. 4c,d). These results were confirmed by the observation that all NPC1- and NPC2-deficient primary fibroblasts displayed reduced infection with CHIKV La Réunion (Fig. 4e). In accordance with the results obtained with normal skin fibroblasts treated with imipramine, NPC1- and NPC2-deficient primary fibroblasts transduced with CHIKV-pseudotyped particles were impaired for viral fusion (Fig. 4f). A similar decrease was observed when the cells were transduced with retroviral particles pseudotyped with VSV-G, but not with GALV-envelope glycoprotein (Fig. 4g). The difference correlates with the mode of entry: VSV and CHIKV both enter into their target cells using endocytosis followed by fusion with endosomal membranes while GALV enters by fusion directly at the plasma membrane. These results show that NPC1 or NPC2 deficiency has a strong impact on the entry and/or fusion steps in the CHIKV life cycle.

### Imipramine also exerts antiviral activity against Flaviviruses

**Figure 5:**
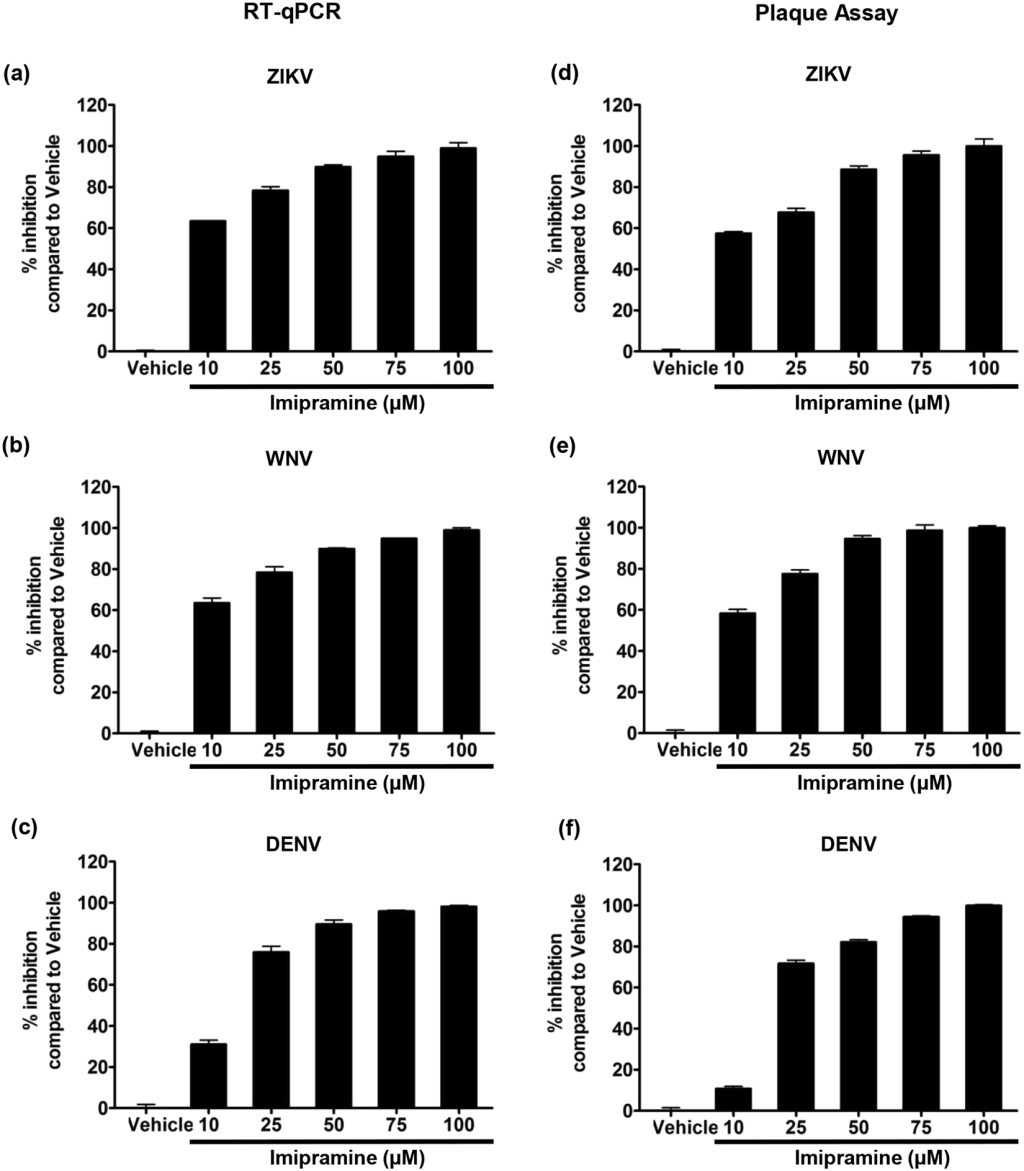
Imipramine inhibits Flavivirus replication in primary human skin fibroblasts. Human skin fibroblasts were pretreated with vehicle or imipramine at the indicated concentrations for 2 h before exposure to different Flaviviruses. Imipramine was present throughout the culture. After 48 h of culture, the presence of virus RNA in cells and infectious virus productions were quantified by RT-PCR and plaque assay, respectively. (**a**,**d**) ZIKV strain Pf13 (MOI 0.1) (**b**,**e**) WNV WT (MOI 0.1) (**c**,**f**) DENV-2 strain 16681 (MOI 0.5). The data represent mean ± SD from three independent experiments.

In order to investigate whether the antiviral effects of imipramine are limited to CHIKV, we extended the evaluation to other arboviruses. In keeping with its antiviral effect on CHIKV, imipramine strongly inhibited, in a dose-dependent manner, the replication of WNV and DENV in human skin fibroblasts as well. It was also active against ZIKV, another member of genus Flavivirus that poses a serious global public health concern. RNA replication and virion production of each virus was effectively inhibited and approached 100% at a concentration of 100 µM (Fig. 5a–f, respectively and Supplementary Fig. S6), without any deleterious effects on cell viability.

## Discussion

The rising global incidence of arboviruses and the absence of adequate antiviral treatment or licensed vaccines underscore the need for the development of novel and effective antiviral compounds. In the present study, we have evaluated whether drugs that interfere with intracellular cholesterol transport have the capacity to inhibit CHIKV replication in epidermal fibroblasts, a major target cell for viral entry in the human host. The results show an antiviral activity of the class II cationic amphiphilic compounds U18666A and imipramine. Both drugs, that induce a phenotype in human fibroblasts reminiscent to that observed in Niemann-Pick type C disease, strongly inhibit the replication not only of CHIKV, but also of several members of the *Flaviviridae* family, including ZIKV, WNV and DENV without any secondary cytotoxic effects.

Cholesterol plays an important role at multiple stages during the infection cycle of a variety of viruses. It has been reported that depletion of cellular cholesterol *in vitro* by methyl-β-cyclodextrine significantly reduces the entry and/or fusion of a broad range of RNA viruses, including members of the Filovirus, Alphavirus and Flavivirus genera^14, 21, 22, 35^ and to some extent of VSV^36, 37^. In addition, various compounds that block the cellular trafficking of cholesterol in the LE/Ls compartment, the site of viral membrane fusion and cytoplasmic escape, have been used to inhibit viral replication in permissive cells. Hence, U18666A was reported to inhibit the entry of DENV and Ebola virus^38–40^ and to block assembly of HIV-1 particles^33, 40^. Our results corroborate these findings and show that U18666A strongly inhibits, in a dose-dependent manner, the replication of CHIKV in human fibroblast cells. This effect is similar to that of imipramine, an FDA-approved antidepressant drug^41^ and is consistent with the results from a previous study showing that imipramine inhibits the production of the Ebola virus in human umbilical vein epithelial cells^35^.

In the present study, imipramine inhibited the entry and/or fusion of retroviral pseudoparticles containing the CHIKV envelope and impaired the post-fusion viral RNA replication steps, as demonstrated by the use of a stable replicon cell line and time-of-addition experiments. The drug therefore seems to interfere with distinct steps of the infectious cycle requiring cholesterol. Indeed, host membrane cholesterol is generally recognized as a key factor for the unmasking of the fusion peptide in class II envelope glycoproteins^42^ including that of CHIKV^14, 43^. Moreover, as intracellular steps of alphavirus replication take place in close association with host membranes, its remains possible that cholesterol may also facilitate CHIKV RNA replication in a way that however remains to be determined.

Like other tricyclic antidepressants, imipramine undergoes biotransformation in the liver, producing progressively more polar metabolites which can then be readily excreted by the kidneys and only a part of imipramine is eliminated without biochemical changes^44^. Pharmacologically active metabolites of imipramine are formed by N -demethylation to desipramine and hydroxylation to 2-OH-imipramine and 2-OH-desipramine, respectively, that are catalyzed by the P450 cytochrome enzymes in the liver. In the present study, we have limited ourselves to the study of the *in vitro* effects of imipramine on skin fibroblasts. However, a study in the literature reports that desipramine blocks cholesterol transport in endosomal membranes showing that this drug, like non-metabolized imipramine, also has a deleterious effect on viral trafficking and replication^45^.

The antiviral effects of imipramine were also conserved for different arboviruses, including the DENV, WNV and ZIKV from genus Flavivirus, thereby attesting for its broad-spectrum antiviral activity. It is expected that these antiviral effects, in addition to a possible direct effect on viral replication, rely on the inhibition of viral fusion with cell membranes as well. Indeed, the structure of the E1 envelope glycoprotein that mediates viral fusion in Alphaviruses is very similar to its counterpart in Flaviviruses, known as Class II viral fusion proteins^42, 46^. Consequently, optimal fusion reactions of DENV and WNV also require cellular cholesterol^21, 22^, accounting for their susceptibility to class II cationic amphiphilic drugs. It is of note that the requirement of cholesterol for the ZIKV life cycle has never been studied thus far, but our results provide a preliminary clue to further explore this pathway.

To further investigate the link between CHIKV infection and intracellular cholesterol trafficking, we used primary fibroblasts from patients with NPC-disease, a lysosomal storage disorder, characterized by an accumulation of cholesterol and other lipids within the LE/Ls. CHIKV infection of these cells, bearing various mutations in the NPC-1 or NPC-2 proteins, resulted in greatly reduced numbers of virus infected cells and diminished virus production. Moreover, transduction of these cells with CHIKV-glycoproteins pseudotyped particles clearly demonstrated that both NPC-1 and NPC-2-deficiency affect the entry/fusion steps in the CHIKV life cycle. The same apparently applies to VSV; in contrast transduction by GALV-pseudotyped particles that fuse directly with the plasma membrane was not affected by NPC-1 deficiency. These findings underscore the notion that NPC-1 acts in a cooperative manner with NPC-2 to traffic cholesterol within LE/Ls^23^. It is as yet unclear how the loss of function of NPC-1 or NPC-2 impacts on viral fusion. Results from several studies have shown that NPC-1 plays a crucial role in filovirus and HIV-1 replication^33, 39^. Indeed, the presence of this protein was shown to be required for membrane fusion mediated by filovirus glycoproteins and viral escape from the vesicular compartment, independent of its known function in cholesterol transport^39^.

It is important to note that cholesterol accumulation may also indirectly affect virus infection, for example by modulating receptor expression or up- or down regulation of the production of proteins with anti- or proviral activity. Other modes or action might be via a change in the composition of membranes where CHIKV RNA replication complexes are formed or where complexes of CHIKV E-proteins are assembled. Finally, higher cholesterol content may also affect the properties of progeny virions themselves, leading to the production of less or even non-infectious viral particles.

## Conclusion

Although the exact steps in the CHIKV replication process affected by imipramine remains to be identified, the data presented here indicate that this kind of compounds could be effective, both at preventing infection of new target cells, as well as at decreasing the replication efficiency in cells already infected by the virus. Its possible use in antiviral treatment in infected patients however is conditioned by several limitations, such as potential side effects and the achievement of an effective dose of the drug in *in vivo* conditions. The limits of the results pertaining to the clinical use of imipramine in antiviral treatment notwithstanding, the present study describes a strategy to help design new antiviral compounds that interfere with cholesterol transport for the treatment of CHIKV and other arbovirus infections.

## Materials and Methods

### Cells and virus

C6/36 *Ae*. *albopictus* cells, used for propagation of the CHIKV strains, were grown at 28 °C in Dulbecco’s modified Eagle’s medium (DMEM; Invitrogen, Cergy Pontoise, France) supplemented with 10% fetal calf serum (FCS; Lonza, Basel, Switzerland) at 28 °C, as previously described^47^. The HFF1 skin fibroblast cell line (ATCC, Manassas, VA) and Vero cells (African green monkey kidney-derived cells, provided by P. Desprès from the Pasteur institute of Paris) were maintained in DMEM supplemented with 15% and 5% FCS, respectively. Fibroblasts from healthy donors (GM09503, GM00500) as well as from patients carrying mutations in the NPC1 (GM00100 (1 A), GM17921 (1B), GM23162 (1 C), GM18397 (1D)) or NPC2 (GM18455 (2 A), GM17910 (2B), GM18429 (2 C), GM18424 (2D)) genes were purchased from Coriell Repositories (Coriell Institute for Medical Research, Camden, NJ). They were maintained at 37 °C, 5% CO_2_ in DMEM supplemented with 5% FCS. The CHIKV replicon cells were cultivated in DMEM (Life Technologies, Saint-Aubain, France) supplemented with 10% FCS, 1% penicillin-streptomycin, 1% of non essential amino acids (Life Technologies) and incubated at 37 °C with 5% CO_2_.

The low-passage-number of the LR2006_OPY1 strain (a kind gift from Dr Philippe Desprès, PIMIT, Inserm U1187, St Clotilde), was isolated from a viremic patient in La Réunion Island in 2006. The DENV-2 strain 16681^48^ and the Israeli WNV strain IS-98-ST1 (WNV_IS98_)^49^ were used in this study. The clinical isolate PF-25013-18 of ZIKV has been previously described^47^. All viruses were grown in C6/36 cells and the experiments were performed at the IRD level 3 biocontainment laboratory facility.

### Antibodies and reagents

The high specificity rabbit anti-CHIKV nsP1, nsP2 and nsP3 antisera, prepared in-house^50^ and previously used for detection of CHIKV ns-proteins upon use of virus inhibitors^51^ were used for the detection of CHIKV nonstructural proteins. Mouse anti-CHIKV envelope 3E4 Cy3- or Alexa Fluor 488-conjugated antibodies were kindly provided by Dr Philippe Desprès. Rabbit anti-NPC1, NPC2 and LAMP-1 antibodies were purchased from ABCAM (Paris, France). Secondary antibodies (HRP conjugated goat anti-mouse or -rabbit) were purchased from Jackson Laboratories (Westgrove, PA). Mouse monoclonal anti-β –actin antibody, U18666A (3 β-[2-(diethylamino)ethoxy]an- drost-5-en-17-one), imipramine hydrochloride, filipin and thiazolyl blue tetrazolium bromide (MTT) were from Sigma-Aldrich (St Louis, MO).

### Viability assay

Cell viability was determined using MTT-based assay. Briefly, cells were treated with different compounds and incubated at 37 °C, 5% CO_2_, washed by phosphate-buffered saline (PBS) and then incubated with 100 µL MTT. After 2 h, MTT was removed and 50 µL of DMSO were added to each well and mixed thoroughly. The mixture was incubated at 37 °C for 10 min and cellular viability was determined measuring the absorbance value at 570 nm.

### Viral Infection

Human fibroblasts were seeded in six-well plates and grown to a 70–80% confluence. The cultures were rinsed twice with PBS and the cells were incubated with CHIKV, DENV, WNV and ZIKV at the desired MOI for 2 h at 37 °C while gently agitating the plates. Then, the inoculum was removed and the cells were washed three times with PBS. DMEM supplemented with 15% FCS was added to each well and the plates were incubated at 37 °C and 5% CO_2_, for the duration of the experiment. Cells serving as a negative control were incubated with culture supernatant from uninfected C6/36 cells.

### CHIKV replicon cell line based assay

Huh-7 cells stably transfected with the CHIKV La Réunion-NCT (Non Cytotoxic) replicon^52, 53^ were used to test the effect of imipramine on CHIKV RNA replication. CHIKV replicon cells were plated in six-well plates, treated with increasing concentrations of imipramine and incubated at 37 °C and 5% CO_2_. After 48 h of incubation, Renilla Luciferase (*Rluc*) activity, expressed by the CHIKV replicon, was detected using the Renilla Luciferase assay (Promega, Charbonnière, France). The luminescence signal, proportional to the CHIKV’s RNA replication, was then measured using the *Modulus microplate luminometer* (Turner BioSystems, CA) and plotted to determine the antiviral activity of imipramine. Vehicle-treated cells were used as a control. A sigmoidal curve fit with variable slope was created to obtain the half maximal effective concentration (EC_50_) value using Graph Pad Prism 5 (Graph Pad Software Inc., San Diego, CA).

### Cholesterol staining

Cells were stained by filipin as previously described^33^. Briefly, cells were fixed with paraformaldehyde (2%) for 15 min at room temperature, washed three times with PBS and incubated with filipin for 30 min at 4 °C. Cells were collected, washed three times with PBS and analyzed by fluorescence microscopy in the DAPI channel.

### Cell staining and immunofluorescence assay

CHIKV-infected cells were fixed with 2% paraformaldehyde for 30 min at RT and then permeabilized with 0.1% saponin for 15 min at 4 °C. The cells were incubated with the Cy3-conjugated anti-CHIKV antibody 3E4 in the presence of 0.1% saponin for 1 h at 4° C. The cells were then washed three times with PBS and stained with filipin. Stained cells were mounted onto glass slides using ProLong antifade mounting media (Molecular Probes, Eugene, OR) and fluorescence was analyzed by Leica microscope. Percentage of colocalization was analysed by JACoP (http://rsb.info.nih.gov/ij/plugins/track/jacop.html) using ImageJ program.

### Plaque assay

Vero cells, grown to 70–80% confluence, were incubated with four separate, ten-fold, dilutions of viral supernatant in DMEM at 37 °C for 2 h. Then, a mix of nutriment solution with agar (Lonza) was added and the cells were maintained at 37 °C for 6 days. For plaque counting, the cells were incubated with 3.7% formaldehyde and 0.5% Crystal violet in 20% ethanol.

### U18666A and imipramine treatment

Human fibroblast cells were pre-incubated with vehicle or increasing concentrations of imipramine or U18666A for 2 or 24 h, respectively. Then, treated cells were infected with CHIKV for 24 h or 48 h before further analysis. For imipramine treatment, the cells were maintained with the drug throughout the infection.

For time-of-addition assay cell cultures were infected with CHIKV at MOI of 10; imipramine was use at concentrations 10 µM, 25 µM, 50 µM, 75 µM or 100 µM and DMSO was used as vehicle control. Cells were pre-treated for 2 h with the inhibitor that was either discarded prior to infection with CHIKV (pre-treatment only) or, alternatively, was maintained in the cell culture throughout the infection. In other setups, the compounds were added together with virus or at 6 hpi and were present until cells were harvested at 48 hpi. The amounts of released virions were determined using plaque titration in Vero cells.

### Western blotting analysis

Cells were lysed on ice in RIPA buffer (150 mM NaCl, 5 mM β-mercaptoethanol, 1% NP-40, 0.1% sodium dodecyl sulfate, 50 mM Tris-HCl, pH 8) supplemented with protease inhibitor cocktail solution (Sigma). The protein concentration was determined by bicinchoninic acid (BCA) assay (Thermo Scientific, Saint Herblain, France). Equal amounts of proteins were mixed with Laemmli sample loading buffer, heated for 5 min at 100 °C, subjected to SDS-PAGE and electrotransferred onto a nitrocellulose membrane. The membrane was blocked with 0.05% Tween 20 in PBS (PBST) containing 5% skim milk for 1 h at RT, incubated overnight at 4 °C with desired primary antibody, washed three times with PBST, and subsequently incubated for 1 h at RT with horseradish peroxidase-coupled secondary antibodies (Cell Signaling, France) in PBST. The membrane was washed three times, and proteins were detected by chemiluminescence using a SuperSignal West Pico chemiluminescent substrate kit (Thermo Scientific). The membrane was then stripped and re-probed with an anti-β-actin to ensure that equivalent levels of protein were loaded in each lane.

### Flow cytometry analysis

Infected cells were trypsinized, washed three times in PBS and infection efficiencies were determined by flow cytometry as previously described^47^. Briefly, cells were fixed with 2% paraformaldehyde, permeabilized using 0.1% saponin, and stained with an anti-CHIKV 3E4 Alexa Fluor 488-conjugated antibody. Stained cells were analyzed using the Becton FACSCalibur flow cytometer (Cell Quest software).

### Viral RNA quantification by real time RT-PCR

Total RNA was extracted from human fibroblasts by using Tri reagent (Sigma, Saint Quentin Fallavier, France). The RNA pellet was resuspended in 25 μl of RNase-free distilled water and stored at −80 °C. The RNA was used for reverse transcription using Moloney murine leukemia virus (M-MLV) reverse transcriptase (Promega, Charbonnieres, France) according to the manufacturer’s instructions. The reaction was carried out using 1 μg total RNA as template for the normalization of viral RNA to the amount of total RNA. The MaximaTM Probe/ROX qPCR Master Mix (2x) (Thermo Scientific) was used in qPCR experiment. Each reaction of 25 μL contained 400 nM of each primer, 200 nM of specific probe and 1x Maxima Probe/ROX qPCR Master Mix. Primers and probes sequences are listed in Supplementary Table S1. The amplification conditions were 95 °C for 10 min followed by 45 amplification cycles of 95 °C for 15 s, 60 °C for 20 s and 72 °C for 30 s. The reactions were performed in an Applied Biosystem 7300 system. Real time data were analyzed using the SDS software (Thermo Fischer Scientific). Viral RNA was quantified by comparing the sample’s threshold cycle (Ct) values with each virus RNA standard curve which was obtained as previously described^47, 54^.

### Production of CHIKV glycoprotein pseudotyped particles

Production of lentivirus particles pseudotyped with CHIKV La Réunion glycoproteins was achieved as previously reported^32^. Briefly, the pCAGGS-EnvCHIKV plasmid encoding 3-E1 region of CHIKV (amino acids 262 to 1248) (a kind gift from Graham Simmons, Blood Systems Research Institute, San Francisco, CA)^55^ or the pCMV-MD-VSV-G and pCMV-MD-GALV plasmids encoding the vesicular stomatitis virus (VSV) glycoprotein G or the Gibon Ape Leukemia virus (GALV) envelope protein, respectively (kindly provided by Jean-Luc Battini, IGMM, UMR5235-CNRS Montpellier) were coexpressed with a GFP reporter pseudogenome in cells stably expressing the HIV-1Gag and Pol proteins. Viral pseudoparticles contained in the culture supernatant were purified by ultracentrifugation, their amounts were normalized according to HIV-1 p24 content measured by ELISA (Innogenetics). Fusion of viral pseudoparticles was monitored by quantification of GFP expression in the target cells. The cells were lysed with RIPA buffer and fluorescence was measured directly from the cell lysate using an Infinite F200PRO fluorometer (Tecan). Values were normalized to the protein content in the sample determined using the BCA Assay (Pierce).

### Data analysis and statistical methods

All data are presented as means ± standard deviation (SD). The student *t* test was used to determine the statistical significance. For all experiments, statistical significance was accepted when at p < 0.05.

## Electronic supplementary material

Supplementary Data
