# Supplementary material for: Imipramine Inhibits Chikungunya Virus Replication in Human Skin Fibroblasts through Interference with Intracellular Cholesterol Trafficking
Source: Sci Rep. 2017 Jun 9;7:3145. doi: 10.1038/s41598-017-03316-5 (PMC5466638; doi:10.1038/s41598-017-03316-5)
Supplement: Supplementary file 1 — Supplementary Data [file 41598_2017_3316_MOESM1_ESM.pdf]

Imipramine Inhibits Chikungunya Virus Replication in Human Skin Fibroblasts through Interference with Intracellular Cholesterol Trafficking

Sineewanlaya Wichit[1], Rodolphe Hamel[1], Eric Bernard[2], Loïc Talignani[1], Fodé Diop[1], Pauline Ferraris[1], Florian Liegeois[1], Peeraya Ekchariyawat[3], Natthanej Luplertlop[3], Pornapat Surasombatpattana[4], Frédéric Thomas[1], Andres Merits[5], Valérie Choumet[6], Pierre Roques[7], Hans Yssel[8,+], Laurence Briant[2,+] and Dorothée Missé[1,*]

[1] Laboratoire MIVEGEC, UMR 224 IRD/CNRS/UM1, Montpellier cedex 5, 34394, France

[2] Centre d'Étude d'Agents Pathogènes et Biotechnologies pour la Santé, CNRS-UMR 5236/UM, Montpellier cedex 5, 34293, France

[3] Department of Microbiology and Immunology, Faculty of Tropical Medicine, Mahidol University, Bangkok, 10400, Thailand

[4] Department of Pathology, Faculty of Medicine, Prince of Songkla University, Songkla, 90110, Thailand

[5] Institute of Technology, University of Tartu, Tartu, 50411, Estonia

[6] Environment and Infectious Risks Unit, Institut Pasteur, Paris, 75015, France

[7] CEA, iMETI, Division of Immuno-Virology, Université Paris Sud and Center for immunology of viral infections and autoimmune diseases Inserm, UMR 1184, Fontenay-aux-Roses, 91190, France

[8] Centre d'Immunologie et des Maladies Infectieuses, Inserm, U1135, Sorbonne Universités, UPMC, APHP Hôpital Pitié-Salpêtrière, Paris, 75013, France

* dorothee.misse@ird.fr

+ these authors contributed equally to this work

Supplementary Figure S1

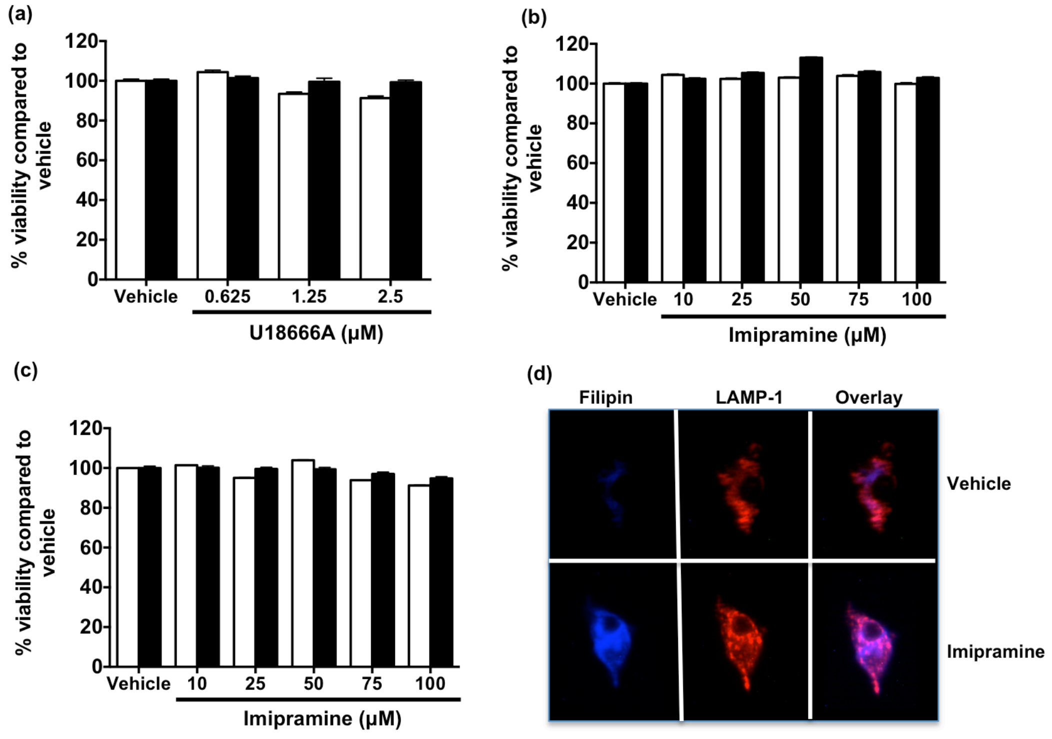

(a) The effect of U18666A on human skin fibroblast viability was quantified by MTT assay; the cells were treated either with water (vehicle) or 0.625, 1.25 and 2.5 µM of U18666A. After 24 h (white bar) and 48 h (black bar), cell viability was determined by MTT assay. (b) Human skin fibroblasts or (c) stable transfected Huh-7 cells with CHIKV-NCT replicon were treated either with PBS (vehicle) or 10, 25, 50, 75 and 100 µM of imipramine. After 24 h (white bar) and 48 h (black bar), cell viability was determined by MTT assay. (d) Stable transfected Huh-7 cells with CHIKV-NCT replicon was treated by vehicle or imipramine for 24 h before fixation and labeling by filipin (blue) or LAMP1 (Red). Colocalization of cholesterol and LAMP1 are shown in violet.

Supplementary Figure S2

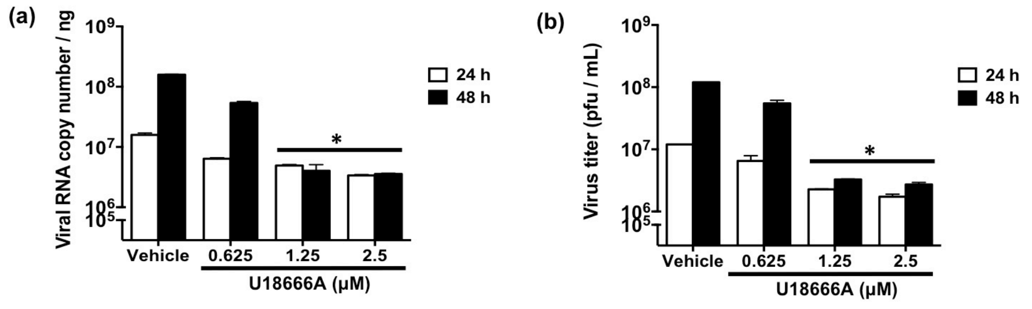

U18666A inhibits CHIKV replication in human skin fibroblasts. Human skin fibroblasts were treated with vehicle or U18666A for 24 h before infection with CHIKV La Réunion strain (MOI 1). After 24 and 48 h, (a) virus RNA and (b) infectious virus production were measured by real time RT-PCR and plaque assay, respectively. The data represent mean ± SD from three independent experiments. *, $p < 0.05$ when compared to cells treated with vehicle.

Supplementary Figure S3

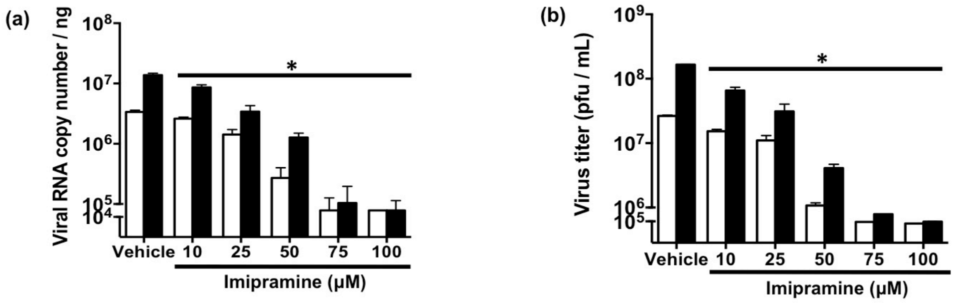

Human skin fibroblasts were pretreated by vehicle or indicated concentrations of imipramine for 2 h before exposed to CHIKV (MOI 1) La Réunion strain (24 (white bar) and 48 h (black bar)). Virus RNA synthesis (a) and infectious virus productions (b) were quantified real time RT-PCR and plaque assay, respectively. The data represent mean ± SD from three independent experiments. *, $p < 0.05$ when compared to cells treated with vehicle.

Supplementary Figure S4

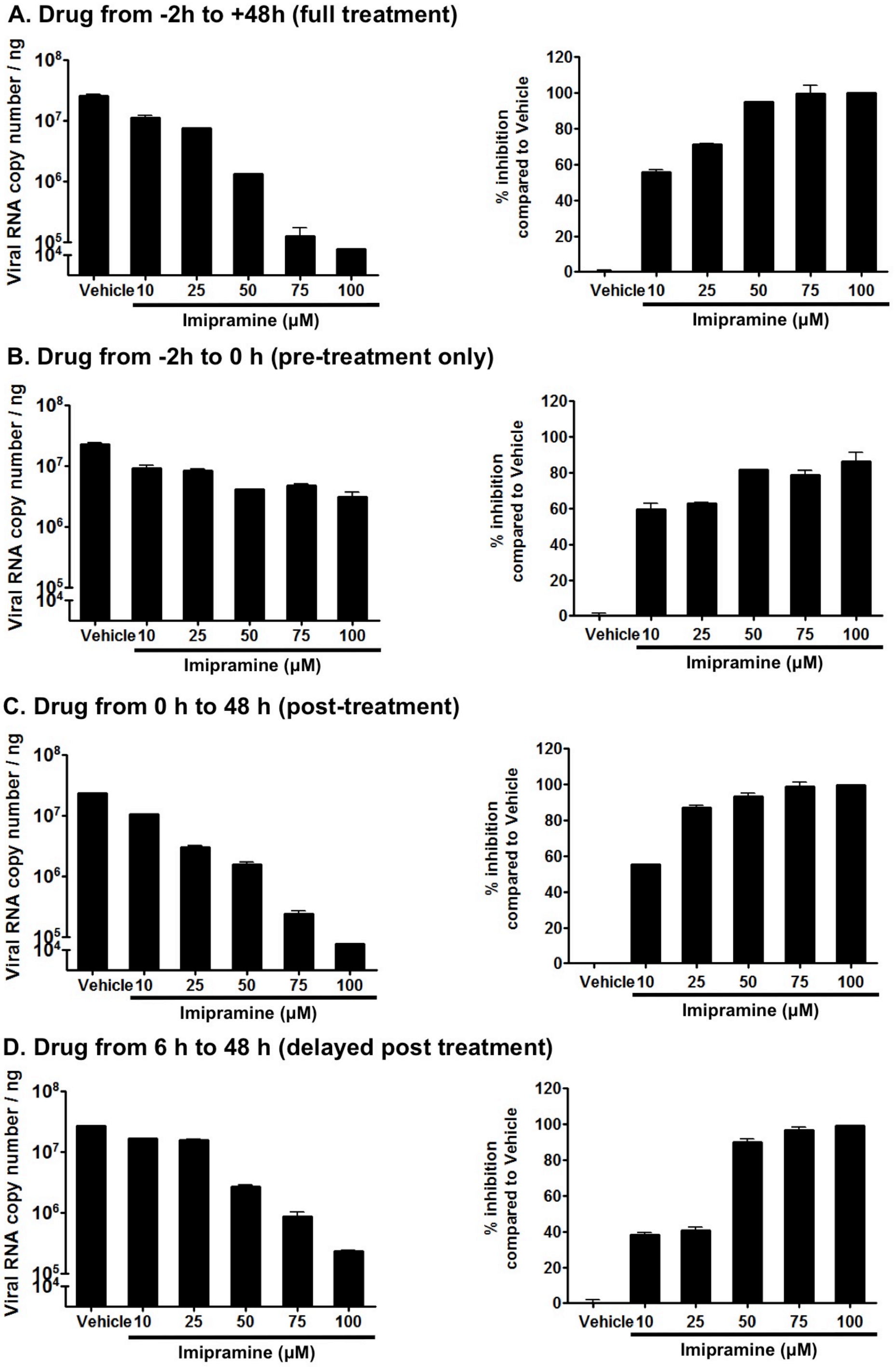

Human skin fibroblasts were treated by vehicle or indicated concentrations of imipramine at different time points. Then treated cells were exposed with CHIKV La Réunion strain (MOI 10) for 48 h. Viral RNA copy numbers, obtained under different conditions, are shown on the left panel; right panel shows achieved percentage of

inhibition calculated using formula [1-(I/V)]*100 where V and I designate experimental values (RNA copy numbers or plaque numbers) at the presence of vehicle and inhibitor, respectively. The data represent mean ± SD from three independent experiments.

Supplementary Figure S5

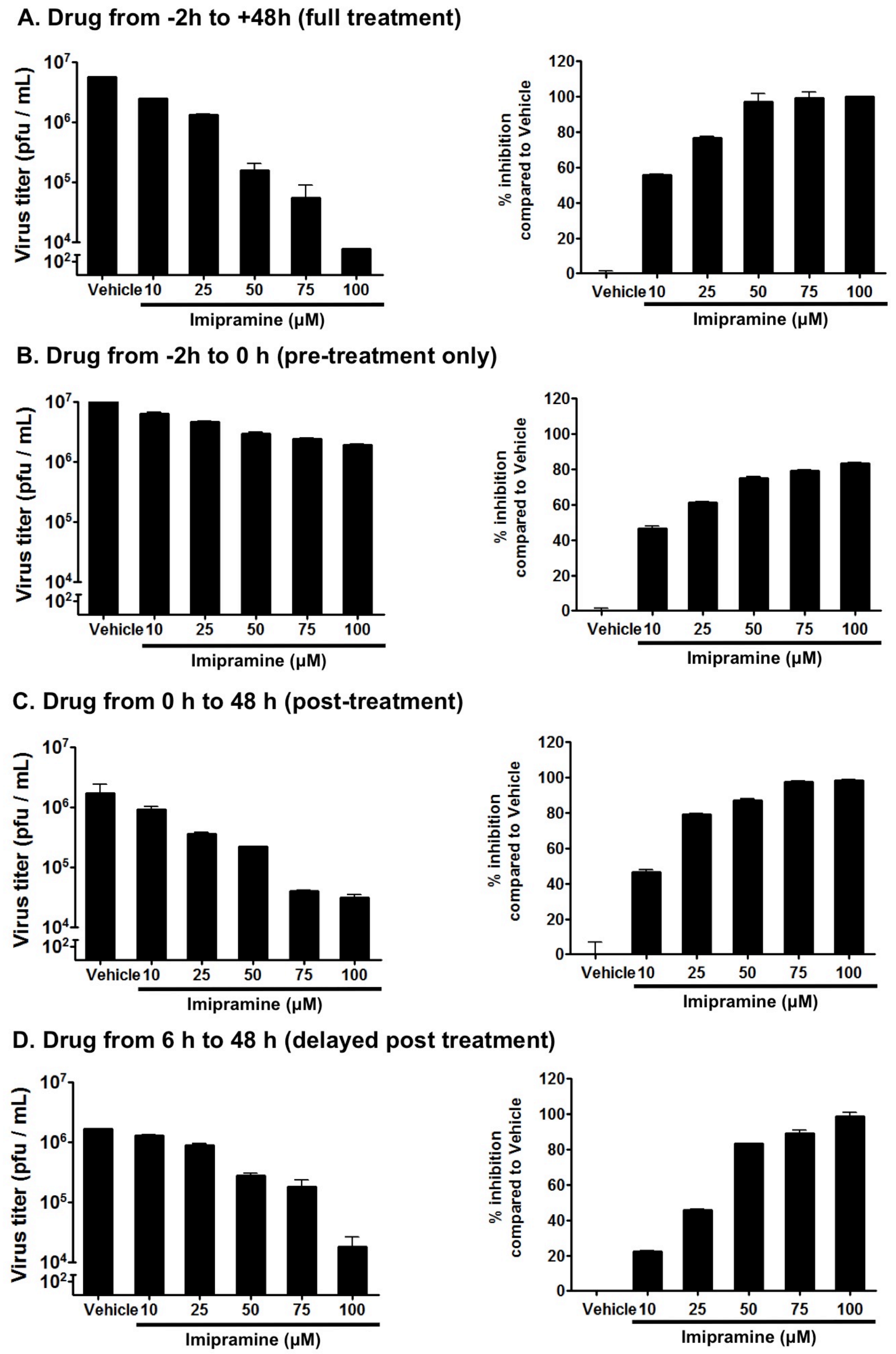

Human skin fibroblasts were treated by vehicle or indicated concentrations of imipramine at different time points. Then treated cells were exposed with CHIKV La Réunion strain (MOI 10) for 48 h. Virus titers, obtained under different conditions, are shown on the left panel; right panel shows achieved percentage of inhibition

calculated as for Supplementary Figure S4. The data represent mean ± SD from three independent experiments.

Supplementary Figure S6

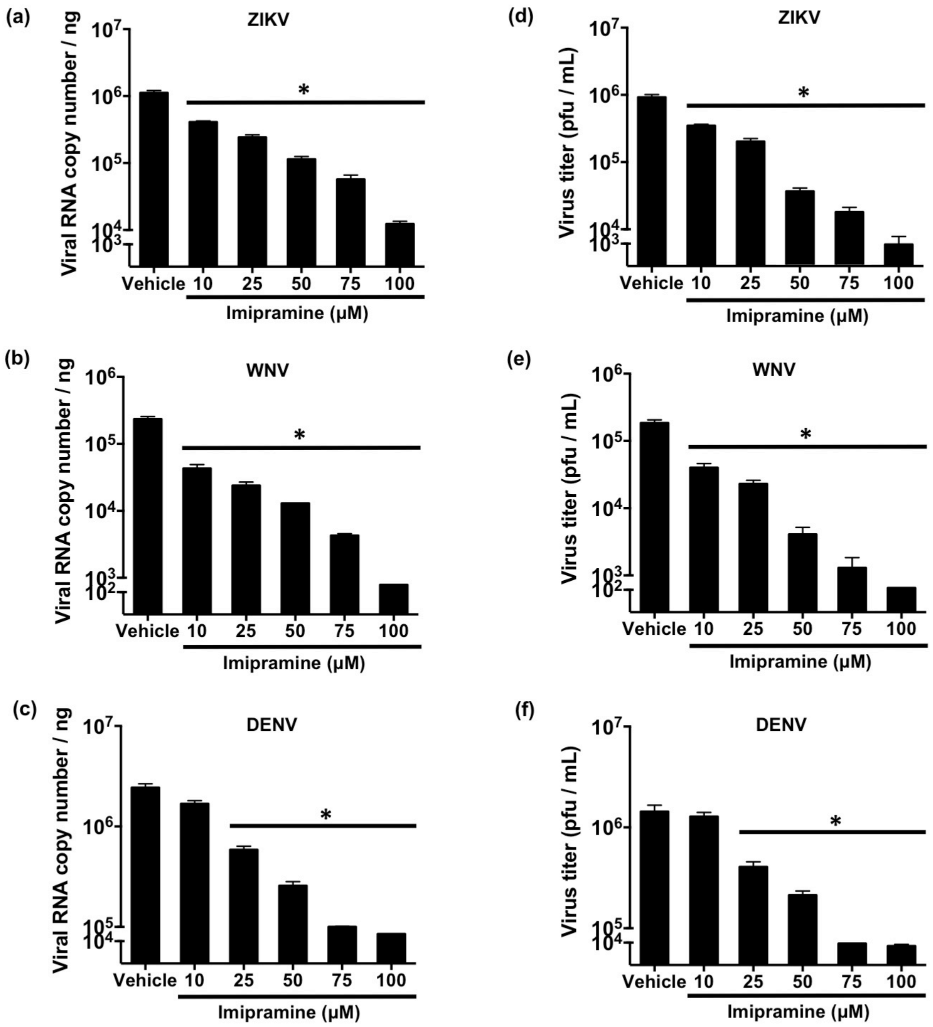

Imipramine inhibits Flavivirus replication in primary human skin fibroblasts. Human skin fibroblasts were pretreated with vehicle or imipramine at the indicated concentrations for 2 h before exposure to different Flaviviruses. Imipramine was present throughout the culture. After 48 h of culture, the amount of virus RNA in cells and infectious virus released to cell culture supernatant were quantified by RT-PCR and plaque assay, respectively. (a and d) ZIKV strain Pf13 (MOI 0.1) (b and e) WNV WT (MOI 0.1) (c and f) DENV-2 strain 16681 (MOI 0.5). The data represent mean ± SD from three independent experiments. *, $p < 0.05$ when compared to cells treated with vehicle.

Supplementary Table S1

Primers and probes for viral detection used in this study.

Virus	Primer	Sequence (5’->3’)
CHIKV	CHIKV-F	5’AAGCT(CT)CGCGTCCTTTACCAAG3’
	CHIKV-R	5’CCAAATTGTCC(CT)GGTCTTCCT3’
	CHIKV-P	5’CCAATGTC(TC)TC(AC)GCCTGGACACCT3’
WNV	WN-LCV-F1	5’GTGATCCATGTAAGCCCTCAGAA3’
	WN-LCV-R1	5’GTCTGACATTGGGCTTTGAAGTTA3’
	WN-LCV-SRH	5’GAAGGAGGACCCCACATGTTG3’
ZIKV	ZIKV-F	5’TTGGTCATGATACTGCTGATTGC3’
	ZIKV -R	5’CCTTCCACAAAGTCCCTATTGC3’
	ZIKV -P	5’CGGCATACAGCATCAGGTGCATAGGAG3’
DENV	DENV-F	5’GGAAGGAGAAGGACTGCACA3’
	DENV -R	5’ATTCTTGTGTCCCATCCTGCT3’
	DENV -P	5’TAAGAGACGTGAGCAAGAAAGAGGGAGGAG3’
